# Effect of Psychological Resilience on Posttraumatic Growth Among Midwives: The Mediating Roles of Perceived Stress and Positive Coping Strategies

**DOI:** 10.1002/nop2.70076

**Published:** 2024-11-01

**Authors:** Yu Zheng, Xin Wang, Yuan Deng, Jia Wang

**Affiliations:** ^1^ Department of Obstetrics Chengdu Fifth People's Hospital Chengdu China; ^2^ Chengdu Women's and Children's Central Hospital, School of Medicine University of Electronic Science and Technology of China Chengdu China; ^3^ Sichuan Provincial People's Hospital, School of Medicine University of Electronic Science and Technology of China Chengdu China

**Keywords:** coping styles, mediating effects, midwifery, perceived stress, posttraumatic growth, psychological, resilience

## Abstract

**Aim:**

To explore the relationship between midwives' psychological resilience and posttraumatic growth, and the mediating role of perceived stress and positive coping strategies.

**Design:**

A multicentre cross‐sectional survey was used.

**Review Methods:**

Data were conducted among 339 midwives from 24 Grade III A hospitals in Sichuan Province from April to July 2023, using the Chinese version of the Connor Davidson Resilience Scale, Chinese version Perceived Stress Scale, Simplified Coping Style Questionnaire, Chinese‐Posttraumatic Growth Inventory and General Demographic Data Questionnaire. A descriptive statistical approach, Pearson's correlation analysis and the Mplus 8.3 were used to analyse the available data.

**Results:**

There was a significant correlation between psychological resilience, perceived stress, positive coping strategies and PTG. Psychological resilience could have a direct positive impact on PTG of midwives, but it could also indirectly affect PTG of midwives through three pathways: the mediating effect of perceived stress, the mediating effect of positive coping strategies and the chain mediating effect of perceived stress and positive coping strategies.

**Conclusion:**

Results suggest that nursing managers should help to improve the psychological resilience of midwives and to reduce individual stress perception, enhance coping abilities and achieve positive growth.

**Impact:**

Prior to data collection, we obtained approval from the nursing department of each hospital. Midwives who met the inclusion and exclusion criteria gave informed consent and invited to participate in this study.

**Reporting Method:**

This study was guided by STROBE.

**Patient or Public Contribution:**

During the data collection phase, 349 midwives took the time to carefully answer the questionnaire items related to this study.

## 1 Introduction

Midwives play a crucial role in assisting mothers to safely deliver healthy newborns. However, the occurrence of traumatic childbirth events during the delivery process may lead midwives to experience grief and emotional challenges (Dai et al. 2021). Traumatic childbirth events refer to incidents that occur during the entire labour process and within a few hours postpartum, which involve actual or threatened severe harm or even death to the mother and/or her baby (Beck 2015; Shorey and Wong 2022). The occurrence of traumatic delivery events such as severe perineal tears, instrumental deliveries, neonatal asphyxia/death and postpartum haemorrhage (Shorey and Wong 2022) not only causes trauma to the mother and her family but may also induce psychological stress reactions in midwives who experience psychological stress as a result of caring for a woman who has experienced a traumatic birth event (Bingham, Kalu, and Healy 2023; Elmir et al. 2017). These reactions may include fear, anxiety, suspicion, guilt, avoidance behaviours, heightened vigilance, and even physical and mental disorders (Dai et al. 2021). Studies have shown that 85.0% of midwives reported having experienced traumatic childbirth events (Bingham, Kalu, and Healy 2023). A survey conducted by Liang et al. (2020) across 12 provinces in China revealed that two‐thirds of midwives had experienced at least three traumatic childbirth events. Despite midwives' extensive professional knowledge and skills, emergencies can occur at any time. In the event of a traumatic childbirth, midwives not only need to make critical clinical decisions in a very short time but also remain calm in highly stressful environments while effectively managing complex situations. This poses an ongoing challenge to their psychological quality and adaptability. Posttraumatic growth (PTG) refers to the positive emotional, cognitive and behavioural changes that individuals experience as they struggle with traumatic events or situations (Tedeschi and Calhoun 2004). Research indicated that the occurrence of PTG significantly reduced the negative symptoms brought by traumatic childbirth events to midwives, such as fear, anxiety and guilt, deepened their understanding of themselves, others, and the world, and positively impacted their physical and mental health (Beck 2015; Sheen, Spiby, and Slade 2016). Currently, empirical research on PTG among midwives is scarce both domestically and internationally. The psychological health of midwives is vital for the quality of midwifery care and the safety of mothers and infants, thus, investigating the factors influencing the PTG among midwives becomes particularly important. Psychological resilience is a psychologically beneficial phenomenon for individual growth and development, enabling individuals to adapt to adversity, recover from stress and transform challenges into opportunities for personal and professional growth (McCoy, Sauer, and Sha 2023). It is a significant factor influencing the PTG. Perceived stress, as an individual's cognitive appraisal of challenging situations, plays a pivotal role in shaping PTG by influencing how one interprets and responds to stress (Zeng et al. 2023). Positive coping strategies, which involve adaptive behaviours and reframing of experiences, have been shown to enhance PTG by promoting emotional recovery and constructive adaptation. These factors are interrelated. Although psychological resilience, perceived stress and positive coping strategies play important roles in the professional lives of midwives, the specific mechanisms through which these variables interact to promote PTG have not been fully studied. This study aims to investigate the mechanisms linking these variables, offering new theoretical support and practical guidance for interventions that support midwives' psychological health, helping them better cope with the stress and foster personal and professional growth.

## 2 Background

Studies have shown that nurses may experience negative psychological impacts and stress reactions after participating in or witnessing traumatic events, but they may also experience positive internal changes and growth (Wang et al. 2022; Joseph and Linley 2008; Okoli and Seng 2021; Yaakubov, Hoffman, and Rosenbloom 2020), and cultivating positive psychology is easier than correcting negative psychology (Tedeschi and Calhoun 2004). Beck, Rivera, and Gable (2017) conducted a mixed‐methods study collecting national registered midwives' PTG data via email. The quantitative study showed that 467 midwives reported lower levels of PTG after experiencing traumatic childbirth events, while the qualitative findings further supported the notion that midwives may experience PTG after such events, as one midwife disclosed, ‘Never doubt the human capacity to overcome and transcend tragedy. The way mothers who have suffered traumatic birth events deal with their trauma is awe‐inspiring and makes us all the more respectful of the human inner strength (Beck, Rivera, and Gable 2017)’.

The occurrence of PTG can help midwives gain positive insights and understanding from negative events in their work, deepening their understanding of themselves, others and the world (Okoli and Seng 2021; Yaakubov, Hoffman, and Rosenbloom 2020; Beck, Eaton, and Gable 2016). Currently, empirical research on midwives' PTG is relatively scarce both domestically and internationally. How midwives perceive their current situation and their current development status, whether they are coping well or experiencing various traumas, are important questions. As a special group in the nursing team, midwives' mental health plays a crucial role and holds significant meaning for the quality of midwifery work and maternal and infant safety. Therefore, it is worth exploring how to stimulate positive work states in midwives by mobilising their psychological resources and promoting growth from experiences of traumatic childbirth events.

Defined as a positive psychological function combining stability and development (Connor and Davidson 2003), psychological resilience is the ability of individuals to adapt, adjust and recover when facing significant crises, trauma or adversity, allowing individuals to maintain relatively stable emotional and psychological states under pressure (McCoy, Sauer, and Sha 2023). The mechanisms underlying psychological resilience are diverse. For instance, within the same stressful context, some individuals employ internal cognitive regulation to interact with the current situation, thereby alleviating stress. Conversely, others rely on external beneficial factors, such as harmonious family atmosphere, intergenerational equality and stable interpersonal relationships, to help individuals reduce fear of the environment, leading to favourable adaptation outcomes (Troy et al. 2023). When an individual's intrinsic protective traits fail to exert their intended effect, external sources such as psychological support can aid in restoring psychological equilibrium. Similarly, when external protective factors alone cannot address hazardous elements, the positive forces of internal factors can compensate for this imbalance (Pinar, Yildirim, and Sayin 2018). Research by McGowan and Murray (2016) indicates that good psychological resilience is a crucial psychological attribute enabling midwives to adapt effectively and achieve positive growth in challenging work environments.

Furthermore, supportive work environments and team dynamics facilitate midwives in better coping with the pressures and challenges of their profession (Feng 2021). Studies have shown that the higher the psychological resilience of midwives, the greater the level of PTG they experience, as trauma‐related challenges to core beliefs can foster personal growth (Beck, Rivera, and Gable 2017; Peng et al. 2021). Additionally, research has demonstrated that psychological resilience is an important factor in predicting the level of PTG (Atay et al. 2022; Liu, Ju, and Liu 2021), with psychological resilience significantly and positively influencing PTG (Feng 2021; Huang et al. 2021). Both psychological resilience and PTG are vital psychological resources for midwives, helping them maintain professional enthusiasm and deliver high‐quality midwifery services. However, there is currently a lack of systematic research on the specific impact of psychological resilience on PTG among midwives, particularly regarding the pathways through which this influence occurs. This study aims to explore the specific mechanisms linking psychological resilience and PTG from a positive psychology perspective, clarifying the relationship between the two and their implications for midwives' professional growth. The study will not only enrich the existing literature but also provide valuable references for nursing managers to develop interventions aimed at enhancing psychological resilience and promoting positive professional growth in midwives faced with traumatic events.

Previous research on the relationship between psychological resilience and PTG has primarily focused on populations such as cancer patients, frontline nurses during the pandemic and earthquake survivors with limited validation among midwives. Studies have found that psychological resilience not only directly influences PTG but also may indirectly affects it through other mediating factors (Wan et al. 2023; Liu, Ju, and Liu 2021; Chen et al. 2022). In the process of indirect influence, perceived stress and positive coping strategies are two important mediating variables worth considering. The Cognitive Phenomenological Transactional (CPT) model of stress posits that when stressors act on individuals, whether the outcome of the stress response is positive (e.g., PTG) or negative depends mainly on the individual's cognitive evaluation and response to the stressor (Lazarus and Folkman 1984). Perceived stress refers to the cognitive evaluation individuals make when facing stressors and stressful situations, which determines the coping behaviours individuals adopt to maintain internal balance (Lazarus and Folkman 1984). Coping, as an internal factor, along with external potential protective factors such as external coping resources and social support, generates stress response behaviours. Problem‐focused coping strategies (positive coping strategies) help buffer psychological distress caused by stressors (Jiang et al. 1993). A study by Chen, Wang, and Zhao (2023) and Chen et al. (2023) demonstrated that nursing students, when facing academic pressure and employment difficulties, tend to adjust their existing cognitive patterns to match the current stress situation and preferentially adopt positive responses and correct attributions to achieve personal growth and inner satisfaction. Similar to the results of Salami et al.'s study on nurses' perceived stress and coping, the process of perceived stress assessment significantly influences coping behaviours, with perceived stress negatively predicting positive coping strategies (Salami, Mozaffari, and Mohammadi 2023).

The Quality‐Stress Interaction Model considers psychological quality as the independent variable, cognitive appraisal, external coping resources, and coping as mediating variables, and disease, adaptation or growth as outcome variables (Liang 2012). The results depend on the severity of the stressor, psychological quality, intuitive assessment and effectiveness of coping, as well as the protective effects of external support and coping resources. Research by Piotrowski et al. (2022) indicates that midwives with higher levels of psychological resilience perceive lower levels of work stress. Perceived stress directly affects individuals' internal states, with a study showing a significant negative correlation between perceived stress and posttraumatic growth (Yaakubov, Hoffman, and Rosenbloom 2020). Ren's study found that positive coping strategies promote psychological resilience, and positive and effective coping strategies can mitigate the negative impact of perceived stress on nurses (Ren et al. 2018). When individuals can effectively cope with stressful events or situations, growth occurs, leading to noticeable psychological maturity and development over time (Finstad et al. 2021). Furthermore, other studies have shown that when stress perception and positive coping styles are considered at the same time, there is a chain mediation effect between them (Chen, Wang, and Zhao 2023; Chen et al. 2023; Yao 2022). Therefore, based on the support of the above theories, this study hypothesises whether perceived stress and positive coping strategies play a role in the relationship between psychological resilience and PTG in midwives, and in what way.

In summary, this study was designed to target midwives who experience psychological stress as a result of caring for a woman who has experienced a traumatic birth event in their work. Drawing from the perspective of positive psychology and integrating the CPT model (Lazarus and Folkman 1984) and the Quality‐Stress Interaction Model (Liang 2012), the study explored whether psychological resilience of midwives influenced PTG. Additionally, perceived stress and positive coping strategies were introduced as mediating variables between psychological resilience and PTG, aimed to address how psychological resilience of midwives impacted PTG.

**FIGURE 1:**
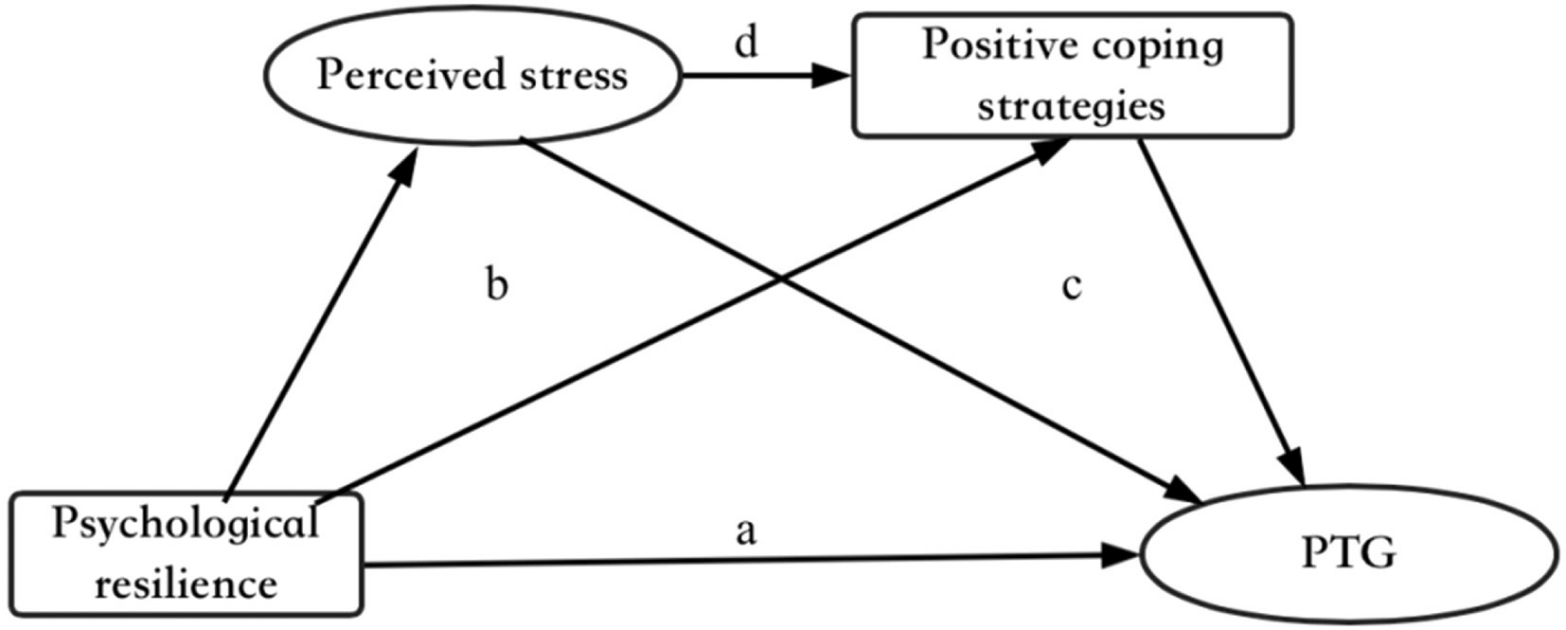
Hypothesised relationships among psychological resilience, PTG, perceived stress, and positive coping strategies.

The relationships hypothesised in the study are shown in Figure 1: (a) psychological resilience would have a positive effect on PTG; (b) perceived stress partially mediated the relationship between psychological resilience and PTG; (c) positive coping strategies partially mediated the relationship between psychological resilience and PTG; (d) perceived stress and positive coping strategies had a serial mediation effect between psychological resilience and PTG.

## 3 Methods

### 3.1 Aims

This study was conducted to investigate the influence of psychological resilience on the PTG of midwives. A structural modelling equation was used to clarify the relationship between psychological resilience and PTG assess the chain mediating effect of perceived stress and positive coping strategies.

### 3.2 Study Design

A multicentre cross‐sectional survey design was conducted to collect data, selecting midwives who met the inclusion and exclusion criteria from 24 Grade III A hospitals in Sichuan Province. Reporting of the study followed the Strengthening the Reporting of Observational Studies in Epidemiology (STROBE) guidelines.

### 3.3 Participants

Midwives who participated met the following inclusion criteria: (a) Holds both a nursing practice qualification certificate and a qualified certificate in maternal and child health care technology; (b) worked as a midwife in the delivery room of a tertiary Grade III A hospitals for over 1 year; (c) possesses independent midwifery capabilities; (d) in the two consecutive years prior to formal investigation (hereinafter referred to as the ‘recent two years’), provided midwifery services such as antenatal, delivery and postpartum care to pregnant women, and was participated in or witnessed traumatic childbirth events in the past 2 years (such as newborn and/or maternal deaths, shoulder dystocia, instrumental delivery, foetal distress, neonatal resuscitation, third‐degree or higher perineal tears and postpartum haemorrhage) and their painful experiences; (e) voluntarily participating in the survey with informed consent.

Exclusion criteria included (a) participants on sick leave, maternity leave, extended leave for personal reasons or engaged in long‐term study or business trips during the survey period; (b) midwives undergoing further midwifery training; (c) individuals who have experienced significant personal or family‐related major events in the past 2 years (such as divorce, loss of a family member, severe illness or mental illness).

According to the method of estimating sample size for multivariate correlation studies, 10 times the number of included variables was included (Ni, Chen, and Liu 2010), considering that this study involved 25 variables, Therefore, we aimed to collect at least 300 valid questionnaires.

### 3.4 Instruments

#### 3.4.1 Demographic Data Questionnaire

Demographic data of the participant was designed by the researchers and included 14 items on gender, age, nation, hospital category, professional title, employment status, number of night shifts per month, years of experience in midwifery, education level, professional background, marital status, average monthly income, whether they are an only child in family and the number of times witnessing or participating in childbirth trauma in the past 2 years.

Four main instruments were used to measure central concepts in this research. These instruments collectively assess how midwives cope with stress and challenges in their work, as well as the positive psychological changes they experience psychological stress as a result of caring for a woman who has experienced a traumatic childbirth event in their work. The Chinese versions of these scales have undergone extensive localisation and validation in previous studies, ensuring their applicability and accuracy in the Chinese setting.

#### 3.4.2 The Chinese Version of the Posttraumatic Growth Inventory (C‐PTGI)

Developed by Tedeschi and Calhoun (1996), culturally translated by Wang (2011), the C‐PTGI is a self‐report scale. It comprises five dimensions: new possibilities, relating to others, personal strength, self transformation and appreciation of life, totalling 20 items. Each item is scored on a Likert 6‐point scale ranging from ‘I did not experience this change as a result of my crisis’ to ‘I experienced this change to a great degree as a result of my crisis’, scoring ranges from 0 to 5, with a total score range of 0 to 100. A total score of ≥ 60 and < 66 suggests a moderate level of PTG, while ≥ 66 indicates a high level (Wang 2011). Higher total scores indicate higher levels of PTG. In this study, the Cronbach's *a* coefficient of the scale was 0.93, and each dimension was 0.75–0.86.

#### 3.4.3 The Chinese Version of the Connor–Davidson Resilience Scale (The Chinese Version of the CD‐RISC)

Developed by Connor and Davidson (2003) and revised by Yu and Zhang (2007), the Chinese version of the CD‐RISC was a self‐report scale. It comprised 25 items categorised into three dimensions: tenacity, strength and optimism. Respondents assigned ratings to each item on a 5‐point Likert scale, with scores ranging from 1 (Never) to 5 (Almost Always). The scale's maximum possible score is 125, with higher scores indicative of heightened psychological resilience. In this study, the Cronbach's *a* coefficient of the scale was 0.92, and each dimension was 0.72–0.88.

#### 3.4.4 Chinese Version of the Perceived Stress Scale (CPSS)

Developed by Cohen, Kamarck, and Mermelstein (1983) and translated by Yang et al. (2007), the CPSS was a self‐report scale. The CPSS consists of 14 items categorised into two dimensions: sense of tension and sense of losing control. A Likert 5‐point scale (0–4) was employed for scoring, with seven items (items 4–7, 9–10, 13) related to the sense of losing control requiring reverse scoring. The sum of scores for each item yielded the total score (0–56), where higher scores indicated higher levels of perceived stress (Yang et al. 2007). In this study, the Cronbach's *a* coefficient of the scale was 075, in which the dimension of sense of tension was 0.79, and the dimension of sense of losing control was 0.74.

#### 3.4.5 Simplified Coping Style Questionnaire (SCSQ)

Developed by Folkman and Lazarus (1980) and translated by Xie (1998), the SCSQ was a self‐report scale. It consisted of 20 items categorised into two dimensions: positive coping strategies (items 1–12) and negative coping strategies (items 13–20), totalling 20 items. A Likert 4‐point scale ranging from ‘never use’ to ‘use very often’ scored from 0 to 3, respectively. Based on the focus of this study, the positive coping strategies dimension was used to assess the degree of positive coping strategies among midwives. The score range for the positive coping strategies was 0–36, with higher scores indicating a higher degree of positive coping (Xie 1998). In this study, the Cronbach's *a* coefficient of positive coping strategies dimension was 0.89.

### 3.5 Data Collection

Convenience sampling was used to select midwives from 24 Grade III A hospitals in Sichuan province that met the inclusion and exclusion criteria. The survey was administered through an online questionnaire link. Before disseminating the questionnaires, the study secured informed consent and cooperation from the nursing and midwifery departments of each participating hospital. Liaison officers, recruited from the maternity wards of each hospital, were provided with in‐person or online video training to familiarise themselves with the study's objectives and the procedures for questionnaire completion. Midwives who met the inclusion and exclusion criteria of the arrangement gave informed consent and scanned the link of the online questionnaire on site to answer the questionnaire independently. Each IP address was restricted to a single questionnaire submission, and the time allocated for completion was limited to approximately 20 min. Following questionnaire completion, respondents could submit their responses. On the day of data collection, the research team reviewed the completed questionnaires and omitted any deemed invalid. A total of 349 electronic questionnaires were distributed, and 320 valid questionnaires were collected, resulting in a response rate of 91.69%, which met the required sample size for this study.

### 3.6 Data Analysis

Data obtained from the online survey were exported to Excel. Statistical analysis was conducted using SPSS 26.0 and Mplus 8.3 software. Descriptive statistics, including frequencies, percentages, means and standard deviations, were employed to describe midwives' demographic characteristics and their scores across various scales. Independent sample *t*‐tests and one‐way analysis of variance (ANOVA) were used to PTG scores among distinct groups of midwives. Pearson correlation analysis was conducted to scrutinise the interrelationships between variables. A structural equation model was constructed using Mplus 8.3 software. Parameter estimation was conducted through maximum likelihood estimation, with bootstrap resampling employed to assess the multiple mediating effects of perceived stress and positive coping strategies in the relationship between psychological resilience and PTG. A significance level of *α* = 0.05 (two‐tailed) was used; *p*‐value < 0.05 for the results indicated statistical significance.

### 3.7 Ethical Considerations

This study has been approved by the REDACTED. All participants signed written informed consent prior to participating in the study. Participation was entirely voluntary, and participants were informed that they could withdraw from the study at any time without any consequences.

## 4 Results

### 4.1 Socio‐Demographic Characteristics

A total of 320 midwives participated in this study, and most participants were female (99.1%), with ages ranging from 24 to 56 years. In total, 83.1% reported having more than 5 years of experience in midwifery, The distribution of Professional title was mainly divided into primary title (55.6%) and intermediate title (37.2%). The number of times participants had been participated in or witnessed traumatic childbirth events was distributed across three levels, accounting for 43.1%, 23.0% and 31.9%, respectively. The univariate analysis of PTG of midwives with different socio‐demographic showed statistical differences in job titles and average monthly income, and the higher the professional title and income, the better their PTG scores. Additional socio‐demographic characteristics are presented in Table 1.

**TABLE 1:** Analysis of differences in PTG on different socio‐demographic characteristics.

Variables	*N* (%)	*t*/*F*	*p*
Gender			
Female	317 (99.1)	−1.312	0.190
Male	3 (0.9)		
Age (years)			
≤ 25	28 (8.8)	1.279	0.278
26–30	118 (36.9)		
31–40	129 (40.3)		
41–50	40 (12.5)		
51–60	5 (1.6)		
Nation			
The Han nationality	307 (95.9)	−1.288	0.199
Minority nationality	13 (4.1)		
Hospital category			
Grade A Maternal and Child Specialised Hospital	196 (61.3)	−1.469	0.143
Grade A tertiary General hospitals	124 (38.8)		
Professional title			
Primary title	178 (55.6)	3.963	0.009
Intermediate title	119 (37.2)		
Senior title and above	23 (7.2)		
Employment status			
Permanent staff	48 (15)	0.802	0.449
Personnel agent	12 (3.8)		
Off staff contract	260 (81.3)		
Number of night shifts (months)			
≤ 3	38 (11.9)	1.532	0.218
4–7	188 (58.8)		
≥ 8	94 (29.4)		
Years of work in midwifery (years)			
< 5	54 (16.9)	0.579	0.678
5–10	129 (40.3)		
11–20	100 (31.3)		
21–30	30 (9.4)		
> 30	7 (2.2)		
Education level			
Secondary Special School	1 (0.3)	0.247	0.864
Junior college	74 (23.1)		
Undergraduate	243 (75.9)		
Master degree or above	2 (0.6)		
Average monthly income (CNY)			
< 5000	53 (16.6)	4.910	0.008
5000–7999	175 (54.7)		
≥ 8000	92 (28.7)		
The only child			
Yes	118 (36.9)	0.012	0.991
No	202 (63.1)		
Marital status			
Married	240 (75.0)	0.345	0.709
Single	73 (22.8)		
Divorce	7 (2.2)		
Professional background			
Nurse	63 (19.7)	−1.419	0.157
Midwifery	257 (80.3)		
Number of times participating in or witnessing childbirth trauma events in the past 2 years(times)			
1–5	138 (43.1)	2.124	0.121
6–10	80 (25.0)		
≥ 11	102 (31.9)		

### 4.2 The Total Score and Dimension Score of Each Variable Scale

The total scores for PTG, psychological resilience, perceived stress and positive coping strategies were 76.09 ± 9.42, 87.18 ± 7.35, 20.31 ± 3.12 and 28.64 ± 3.93. The scores of each scales and dimensions are shown in Table 2.

**TABLE 2:** Scores of each scales and dimensions.

Scales and dimensions	Entries (number)	Total score ($\bar{x}$ *± s*)	Average score ($\bar{x}$ *± s*)
Psychological resilience	25	87.18 ± 7.35	3.48 ± 0.29
Tenacity	13	44.66 ± 4.28	3.44 ± 0.33
Strength	8	28.67 ± 2.47	3.58 ± 0.31
Optimism	4	13.85 ± 1.40	3.46 ± 0.35
Perceived stress	14	20.31 ± 3.12	1.45 ± 0.22
Sense of tension	7	12.08 ± 2.37	1.73 ± 0.34
Sense of losing control	7	8.23 ± 1.48	1.24 ± 0.22
Positive coping strategies	12	28.64 ± 3.93	2.39 ± 0.33
PTG	20	76.09 ± 9.42	3.81 ± 0.47
Self transformation	4	15.13 ± 1.99	3.78 ± 0.50
Personal strength	3	11.23 ± 1.61	3.80 ± 0.59
Relating to others	3	11.38 ± 1.78	3.74 ± 0.54
New possibilities	4	15.06 ± 2.16	3.77 ± 0.54
Appreciation of life	6	23.29 ± 3.42	3.88 ± 0.57

### 4.3 Correlations Among Variables

Table 3 displays the correlations between variables. Pearson correlation analysis results indicated that PTG was positively correlated with psychological resilience (*r* = 0.679, *p* < 0.01), negatively correlated with perceived stress (*r* = −0.340, *p* < 0.01) and positively correlated with positive coping strategies (*r* = 0.427, *p* < 0.01). Psychological resilience was negatively correlated with perceived stress (*r* = −0.260, *p* < 0.01) and positively correlated with positive coping strategies (*r* = 0.331, *p* < 0.01). Perceived stress was negatively correlated with positive coping strategies (*r* = −0.288, *p* < 0.01).

**TABLE 3:** Correlations among variables.

Variables	1	2	3	4	5	6	7	8	9	10	11	12	13	14
1 Psychological resilience total score	1													
2 Tenacity	0.952**	1												
3 Strength	0.906**	0.771**	1											
4 Optimism	0.747**	0.586**	0.642**	1										
5 Perceived stress total score	−0.260**	−0.246**	−0.224**	−0218**	1									
6 Sense of tension	−0.348**	−0.329**	−0.308**	−0.279**	0.806**	1								
7 Sense of losing control	0.095	0.089	0.094	0.06	0.441**	−0.176**	1							
8 Positive coping strategies total score	0.331**	0.298**	0.295**	0.313**	−0.288**	−0.390**	0.112*	1						
9 Posttraumatic growth total score	0.679**	0.651**	0.615**	0.491**	−0.340**	−0.386**	0.019	0.427**	1					
10 Self transformation	0.549**	0.536**	0.481**	0.397**	−0.222**	−0.256**	0.017	0.323**	0.827**	1				
11 Personal strength	0.619**	0.589**	0.581**	0.429**	−0.334**	−0.393**	0.04	0.405**	0.902**	0.664**	1			
12 Relating to others	0.502**	0.482**	0.439**	0.390**	−0.187**	−0.222**	0.025	0.342**	0.782**	0.621**	0.596**	1		
13 New possibilities	0.650**	0.622**	0.589**	0.475**	−0.348**	−0.395**	0.019	0.386**	0.925**	0.671**	0.821**	0.699**	1	
14 Appreciation of life	0.641**	0.612**	0.591**	0.456**	−0.364**	−0.398**	−0.001	0.413**	0.938**	0.686**	0.871**	0.616**	0.852**	1

*p < 0.05; **p < 0.01.

### 4.4 Structural Equation Modelling Results

**FIGURE 2:**
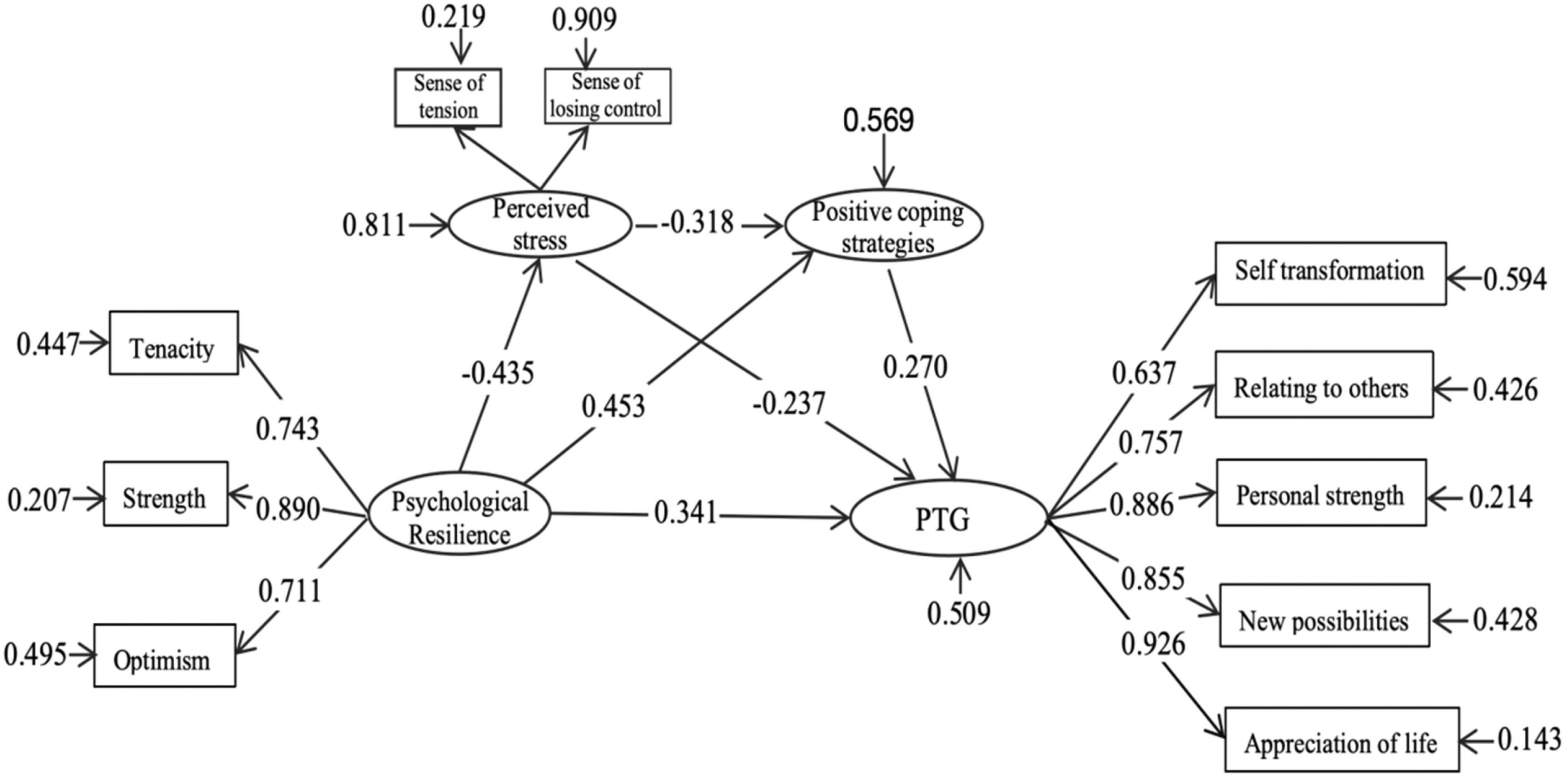
Structural equation model diagram of chain intermediary action.

All data were analysed using SPSS statistics 26.0 and Mplus 8.3 statistical software. A structural equation model was constructed with psychological resilience as the independent variable, PTG as the dependent variable, and perceived stress and positive coping strategies as the mediating variables. Parameter estimation was conducted using the maximum likelihood method. Firstly, the significance of the direct effect path coefficient of psychological resilience on PTG was tested. Demographic variables (professional title, average monthly income) that might influence the results were controlled for. The model fit indices were as follows: *χ* ^2^/df = 1.949, *p* < 0.01, CFI = 0.965, TLI = 0.949, RMSEA = 0.054, SRMR = 0.037, indicating good model fit (Wen and Liang 2015). As shown in Figure 2, psychological resilience positively predicted PTG (*β* = 0.603, *p* < 0.001), demonstrating that the direct effect path coefficient of psychological resilience on PTG was significant, confirming hypothesis a. Next, perceived stress and positive coping strategies were included in the direct effect model for testing their mediating effects. The model fit indices were *χ* ^2^/df = 1.640, *p* < 0.001, CFI = 0.986, TLI = 0.980, SRMR = 0.028, RMSEA = 0.045, indicating good model fit. As shown in Table 4, psychological resilience positively predicted positive coping (*β* = 0.453, *p* < 0.001) and PTG (*β* = −0.341, *p* < 0.001), and negatively predicted perceived stress (*β* = −0.435, *p* < 0.001). Perceived stress negatively predicted positive coping (*β* = −0.318, *p* < 0.001) and PTG (*β* = −0.237, *p* = 0.001), while positive coping positively predicted PTG (*β* = 0.270, *p* < 0.001), as shown in Table 4 and Figure 2.

**TABLE 4:** Coefficient test for each path.

Paths	Effect	SE	*t*	*p*	Boot 95% CI	
					LLCI	ULCI
Psychological resilience → perceived stress	−0.435	0.075	−5.820	< 0.001	−0.581	−0.288
Perceived stress → PTG	−0.237	0.074	−3.119	0.001	−0.382	−0.092
Psychological resilience → PTG	0.341	0.058	5.868	< 0.001	0.227	0.455
Positive coping strategies → PTG	0.270	0.065	4.176	< 0.001	0.143	0.397
Psychological resilience → positive strategies	0.453	0.065	6.993	< 0.001	0.326	0.580
Perceived stress → positive coping strategies	−0.318	0.077	−4.120	< 0.001	−0.469	−0.166

*Note:* Unstandardised regression coefficients reported. Bootstrap sample size 5000.

Abbreviations: CI, confidence interval; L, lower limit; SE, standard error; U, upper limit.

The mediation effects were tested using the bias‐corrected nonparametric percentile bootstrap method (with 5000 resamples). After adding the mediating variables perceived stress and positive coping strategies, the direct effect of psychological resilience on PTG remained significant, with an effect size of 0.341. Additionally, psychological resilience also influenced PTG through three mediating paths: (1) Path 1: psychological resilience → perceived stress → PTG, with an effect size of 0.103, accounting for 17.08% of the total effect; (2) Path 2: psychological resilience → positive coping Strategies → PTG, with an effect size of 0.122, accounting for 20.23% of the total effect; (3) Path 3: psychological resilience → perceived stress → positive coping Strategies → PTG, with an effect size of 0.037, accounting for 6.14% of the total effect. The 95% confidence intervals for the coefficients of the three mediating paths did not include zero, indicating significant mediation effects for all three paths and significant chain mediation effects, confirming hypotheses b, c and d. Therefore, perceived stress and positive coping partially and chain mediated the relationship between psychological resilience and PTG, with a total mediation effect size of 0.262, accounting for 43.45% of the total effect, as shown in Table 5.

**TABLE 5:** Bootstrap analysis of significance test of intermediary effect.

Paths	Effect	SE	Boot 95% CI		Effect proportion (%)
			LLCI	ULCI	
Ind 1: Psychological Resilience → Perceived Stress → PTG	0.103	0.045	0.013	0.191	17.08%
Ind 2: Psychological Resilience → Positive Coping Strategies → PTG	0.122	0.041	0.031	0.201	20.23%
Ind 3: Psychological Resilience → Perceived Stress → Positive Coping Strategies → PTG	0.037	0.014	0.009	0.065	6.14%
Direct effect	0.341	0.058	0.227	0.455	56.55%
Total indirect effect	0.262	0.055	0.153	0.367	43.45%
Total effect	0.603	0.072	0.456	0.750	

Abbreviations: CI, confidence interval; L, lower limit; SE, standard error; U, upper limit.

### 4.5 Common Variance Method Detection

A common variance method detection was conducted using Harman single factor analysis. The results showed that the interpretation rate of the first principal component factor was 23.39% (< 40%) and 16 factors (> 1) with feature roots greater than 1 when not rotated, indicating that there was no serious common method bias in this study (Zhou and Long 2004).

## 5 Discussion

### 5.1 Midwives in Grade III A Hospitals Sichuan Province Exhibit High Levels of PTG

In this study, the total score for PTG among midwives in Grade III A hospitals in Sichuan Province was 76.09 ± 9.42, indicating an overall high level of PTG. This score surpasses the findings of most domestic studies on PTG among nursing professionals conducted by Cui et al. (2020), Zeng et al. (2023) and Fang, Qian, and Wu (2022). Furthermore, it also exceeds the scores from a mixed‐methods study conducted by Professor Beck, Eaton, and Gable (2016) in the United States, which used both quantitative surveys and qualitative interviews to investigate PTG among midwives. Across all dimensions, the scores ranged from 3 to 4 points (slightly above the middle level), suggesting that midwives demonstrate substantial adaptive capabilities and experience positive psychological growth when faced with trauma or challenging events in their work. The results of this study not only provide theoretical support for the existence of PTG but also confirm the prevailing view that PTG is commonly observed in various trauma‐exposed groups. Among the five dimensions, the ‘Personal Strength’ dimension had the highest scores (with an average score of 3.88 ± 0.57), which is consistent with the findings of Cui et al. (2020). This result may be closely related to the nature of midwifery work and the life experiences of midwives. Midwives are directly involved in critical moments of life due to the nature of their profession. This direct involvement allows them to experience life's turning points and significance firsthand. Additionally, their emotional connection with expectant mothers and newborns, coupled with the unpredictability of the childbirth process, enables midwives to gain a deeper understanding of the fragility and preciousness of life. Particularly, when midwives participating in or witnessing traumatic childbirth events, they tend to engage in positive reflection, discovering their intrinsic value and meaning amidst challenges. Consequently, they develop new insights and a deeper appreciation of life. Therefore, nursing managers should provide timely psychological intervention and support to midwives who participate in or witness traumatic childbirth events, thereby promoting their PTG.

The results of this study showed that midwives with higher professional title and higher average monthly income exhibited higher levels of PTG. This may have been because the more senior and experienced midwives have developed a level of resilience to be able to manage their professional lives after experiencing an event that has challenged them, and had access to more social support resources. Additionally, higher professional title and income often came with greater professional recognition and a sense of stability, allowing them to find personal meaning and value when dealing with traumatic events. For midwives with lower professional title, younger age and lower income, their early work experiences may have left them less equipped to cope with traumatic events, making them more susceptible to high turnover rates. To reduce turnover among these midwives, healthcare institutions should have implemented the following measures: First, provide targeted psychological support and training to help them enhance psychological resilience. Second, establish effective mentorship programmes to ensure that younger midwives received guidance and support from more experienced colleagues. Additionally, improving working conditions and offering better career development opportunities could have increased their job satisfaction and reduced the likelihood of leaving the profession.

### 5.2 The Relationship Between Psychological Resilience and Midwives' PTG

The results of this study demonstrate that midwives' psychological resilience positively predicts PTG. In this study, the total score for midwives' psychological resilience was 87.18 ± 7.35, which was above the medium level (75 points higher than the median score of the scale), consistent with the survey results of Chen, Sun, et al. (2020) and Chen, Han, et al. (2020) on the mental resilience of 70,932 nurses from 23 provinces in China. It is possible that this study was conducted after the outbreak of the COVID‐19 pandemic, during which midwives in Grade III A hospitals received emergency training and exercises, which had a positive effect on their psychological stress management and overall psychological resilience. PTG primarily relies on an individual's strengths, and psychological resilience, as a personal trait, is a crucial predictive factor for PTG, in line with previous research findings (Xing et al. 2023; Sun et al. 2023; Atay et al. 2022). Psychological resilience is an essential attribute for nursing professionals to survive and adapt in high‐stress work environments. Midwives with higher psychological resilience are better equipped to cope with emotional challenges and high‐pressure situations in midwifery practice (Duffield et al. 2014). They develop positive coping strategies, establish emotional support networks and maintain an optimistic attitude. These mechanisms help midwives alleviate emotional stress, reduce emotional fatigue and achieve positive growth after experiencing trauma. In this study, one of the dimensions of psychological resilience among midwives, ‘Strength’, which represents an individual's confidence and resilience when facing challenges, had the highest scores (with an average score of 3.58 ± 0.31). This result suggests that midwives tend to approach challenges with a positive attitude, viewing them as opportunities rather than threats (Minooee et al. 2021). They consider their work a highly mission‐oriented profession, and this sense of responsibility motivates them to demonstrate higher levels of strength in dealing with various unexpected situations at work. Consequently, they experience greater satisfaction and growth and are more likely to achieve the ‘Personal Strength’ dimension of PTG.

### 5.3 Analysis of the Separate Mediating Effects of Perceived Stress and Positive Coping Strategies

The results of this study indicate that perceived stress partially mediates the relationship between midwives' psychological resilience and PTG, with perceived stress negatively influencing both psychological resilience and PTG. In this study, the total score for perceived stress among midwives was 20.31 ± 3.12, which is lower than the findings reported by Xu et al. (2021), Shen (2022) and Tian (2022). Specifically, the scores for the ‘sense of tension’ dimension were higher (with an average score of 1.73 ± 0.34) than those for the ‘sense of lossing Control’ dimension (with an average score of 1.24 ± 0.22). Midwifery work involves high‐risk and complex maternal and neonatal delivery scenarios, and some midwives may face the risk of medical accidents due to participating in or witnessing traumatic childbirth events (Beck, Eaton, and Gable 2016). When in such situations, the initial factor in perceiving stress is the unpredictability of childbirth outcomes and the potential for medical liability disputes, leading to a higher score for tension compared to the loss of control (Dai et al. 2021; Shorey and Wong 2022). Nursing managers should thoroughly understand the personality traits and psychological resilience levels of midwives. By helping midwives cultivate a positive mindset and establish a correct self‐concept, they can enhance their psychological resilience (Feng 2021). Additionally, nursing managers should encourage midwives to fully express negative thoughts and release negative emotions, guiding midwives to view traumatic events from a constructive perspective and explore the positive meanings behind these events can reduce their perceived stress levels and promote PTG.

Furthermore, the results of this study reveal that positive coping strategies partially mediate the relationship between midwives' psychological resilience and PTG, with positive coping strategies positively influencing both psychological resilience and PTG. In this study, the average score for positive coping strategies among midwives was 28.64 ± 3.93, indicating a relatively high level of positive coping. Due to differences in individual internal resources, when facing the same stressors, individuals may have varying cognitive evaluations, resulting in different intensities of stress reactions and coping strategies (Nie et al. 2020). Past research has found that coping strategies play a vital mediating role in stress responses and have a significant impact on an individual's physiological and psychological health (Fradelos et al. 2023). In this study, midwives in Grade III A hospitals in Sichuan Province generally tend to adopt positive coping strategies when problem‐solving, which is similar to previous research findings (Yin et al. 2022; Lu et al. 2022). Midwives with higher psychological resilience typically possess effective problem‐solving skills. Those who favour positive coping strategies tend to reevaluate the impact and significance of events and attempt to draw positive experiences from them. They learn how to cope with stress, uncertainty and complexity, thereby reducing the long‐term psychological burden that traumatic events might otherwise bring. This leads to greater insights and personal growth after experiencing trauma.

Therefore, nursing managers should pay attention to the coping strategies chosen by midwives, guiding them to face stress directly and actively seek external help and support. Meanwhile, mutual support and experience sharing among colleagues within the work team can enhance midwives' coping strategies and promote their professional growth (Dai et al. 2021). Additionally, understanding and emotional support from family and friends are equally important, as they not only help maintain midwives' positive emotional well‐being but also offer protection to those experiencing psychological stress from caring for a woman who has experienced a traumatic birth event (Bingham, Kalu, and Healy 2023). On the part of social organisations, efforts should be made to strengthen psychological and emotional support for midwives, closely monitor their psychological changes and offer timely counselling to assist them in recovering and growing after traumatic events (Garcia et al. 2023). This comprehensive support system not only helps to enhance midwives' psychological resilience but also optimises their coping strategies, effectively promoting profound growth and transformation at both professional and personal levels.

### 5.4 Chain Mediation Analysis of Perceived Stress and Positive Coping Strategies

The results of the chain mediation analysis in this study demonstrate that perceived stress and positive coping strategies play a chain mediating role in the relationship between midwives' psychological resilience and PTG. This implies that the level of psychological resilience in midwives in Grade III A hospitals in Sichuan Province not only directly impacts their PTG but also indirectly affects individuals' PTG levels through perceived stress and positive coping strategies. This finding aligns with the CPT Model. Additionally, Yao's study (Yao 2022) on college students provides additional support and validation for the results of this study, indicating that individuals with high psychological resilience tend to perceive less suffering when facing stressful events. They have the ability to quickly adjust their state and are more inclined to adopt positive ways to solve problems, ultimately achieving holistic personal development. In this study, midwives exhibited high levels of psychological resilience, positive coping strategies and PTG, along with low levels of perceived stress. This might be related to the fact that the survey participants were all selected from Grade III A hospitals, where healthcare professionals have better caregiving skills, which can contribute to higher job satisfaction and better psychological well‐being among midwives. High levels of psychological resilience can be seen as a positive cognitive perception of self‐competence, wherein individuals transform stress into positive factors through positive cognitive patterns (Huang et al. 2022). Positive coping strategies can be viewed as proactive behavioural approaches individuals take when facing problems, thereby influencing PTG. Given the significant direct and indirect effects of psychological resilience on PTG among midwives, it is crucial to promote growth in midwives who participating in or witnessing traumatic childbirth events and to prevent these events from affecting their physical and mental health. Midwives should actively recognise themselves, regularly adjust their emotions, and strengthen and expand their social support networks. Simultaneously, nursing managers should focus on midwives with low psychological resilience, high perceived stress and high levels of negative coping, help these midwives develop and cultivate an optimistic, positive and resilient mindset, guide them to cognitively appraise stressful events from a positive perspective, enhance their ability to cope positively with stress and inspire their pursuit of personal growth and development.

### 5.5 Implications for Midwifery

Our findings hold noteworthy implications for the mental health of midwives who participating in or witnessing traumatic childbirth events. First, midwives with high levels of psychological resilience are more likely to achieve PTG. Therefore, nursing managers should focus on the needs and challenges faced by midwives after traumatic events, encouraging them to find deeper meaning in their experiences and enhancing their psychological regulation abilities to improve their levels of PTG. Second, the key finding of the study highlights the chain mediating effect, which indicates that as psychological resilience increases and perceived stress decreases, individuals are more likely to adopt positive coping strategies, ultimately facilitating PTG. Hence, it is essential to provide midwives with education and training to enhance their psychological resilience, helping those in negative emotional states to seek new meanings and values. One effective strategy is encouraging midwives to communicate openly with family and friends, offering them emotional outlets to process their experiences and seek support, which can significantly alleviate stress and maintain emotional well‐being (Bingham, Kalu, and Healy 2023). Additionally, healthcare institutions could incorporate semi‐structured interviews to more deeply explore the inner experiences of midwives who experience psychological stress as a result of caring for a woman who has experienced a traumatic birth event. These interviews would provide insights into their emotional and psychological responses, allowing for more tailored interventions that address their unique challenges. Peer support networks also play a critical role, enabling midwives to share coping mechanisms and strengthen collective resilience (Dai et al. 2021). Therefore, these approaches not only bolster psychological resilience but also provide a comprehensive framework for enhancing both work and life quality for midwives, ultimately leading to personal growth.

### 5.6 Limitations and Future Research Directions

This study has several limitations. First, it employed a cross‐sectional research design to analyse the relationships between variables, observing and measuring participants' data at a single time point. This approach cannot provide information about temporal effects or how variables change over time, thus having inherent limitations. Future research should consider longitudinal study designs to observe changes in individuals or groups at different time points. This would help capture the process of PTG, including details about development, transitions, changes and how individuals adapt to trauma, cope with challenges and build stronger psychological resilience over time. Second, in the study design, the midwives' ‘psychological stress’ factor selected only ‘psychological stress arising from caring for a woman who had experienced a traumatic birth event’. However, ‘psychological stress as a result of caring for a woman who has experienced a traumatic birth event’ is not the only factor contributing to ‘psychological stress’ among midwives. Future studies may adopt a multi‐factor study design to further systematically explore the mechanism of PTG. Third, the results of the study are limited to the mediating role of perceived stress and positive coping strategies in the relationship between psychological resilience and PTG among midwives specifically in the context of childbirth‐related trauma. Future research may consider factors such as personality traits, family environment, self‐reflection and other variables closely related to PTG among midwives. This would allow for a more systematic exploration of the mechanisms underlying PTG.

## 6 Conclusion

In conclusion, this study indicated significant correlation between psychological resilience, perceived stress, positive coping strategies and PTG. Psychological resilience could have a direct positive impact on PTG of midwives, but it could also indirectly affect PTG of midwives through three pathways: the mediating effect of perceived stress, the mediating effect of positive coping strategies and the chain mediating effect of perceived stress and positive coping strategies. Nursing managers should help midwives alleviate perceived stress, enhance their adaptability and boost their confidence in coping with current situations by improving their psychological resilience. This, in turn, can foster a more optimistic outlook on developmental outcomes and ultimately lead to positive growth.

## Author Contributions

Made substantial contributions to conception and design, or acquisition of data, or analysis and interpretation of data: Y.Z., X.W., Y.D. Involved in drafting the manuscript or revising it critically for important intellectual content: Y.Z., X.W., J.W. Given final approval of the version to be published. Each author should have participated sufficiently in the work to take public responsibility for appropriate portions of the content: X.W., Y.D. Agreed to be accountable for all aspects of the work in ensuring that questions related to the accuracy or integrity of any part of the work are appropriately investigated and resolved: Y.Z., X.W., Y.D., J.W.

## Ethics Statement

This study was approved by the Ethics Committee of Chengdu Women and Children's Central Hospital, with approval number: Research Ethics 2023 (17).

## Conflicts of Interest

The authors declare no conflicts of interest.

## Data Availability

The data that support the findings of this study are available from the corresponding author upon reasonable request.
